# Extract of Letter from a Correspondent

**Published:** 1871-08

**Authors:** 


### AN EXTRACT FROM A LETTER FROM AN OLD AND ESTEEMED CORRESPONDENT.

"I have been a sincere and warm admirer of your Journal since its commencement, and have watched its progress with increasing interest and admiration. From the very start you seemed to have caught the true idea of popularizing a Medical Journal, for, instead of compelling your readers to sift the few valuable thoughts from as many articles, you have condensed and culled the cream from a hundred sources, and thrown it in his hand for his immediate use. But the last feature is certainly the crowning one for the promotion of all the best interests of the profession. I allude, of course, to your plan of having all parts of this, and other adjoining States, represented by associate Editors—gentlemen of fine abilities and unquestionable position. The interest of medicine and your Journal will be greatly benefitted by this arrangement.

We all fully, no doubt, appreciate the labors and researches of the able physicians of our larger cities and towns, but in our admiration of these, we have unjustly overlooked the great practical experience and large amount of accumulated facts in the possession of the physicians of our villages and rural districts. You may select any journal in this country, and you will find that the bulk of the finest articles appearing in them are from the pens of physicians of our smaller towns and villages. Gentlemen, yours is a noble effort. Your field is a wide one. It extends into every part of our country; for you labor for the good of humanity, weighted with disease and suffering. Thus far you have nobly acted your part; others now come to aid you with vigorous intellects and noble purposes. Every true heart must bid you entire success in such a holy undertaking. Pursue the even tenor of your way with unshaken confidence in the fact, that the true and scientific men of the profession, will come up "shoulder to shoulder," in defence of those great principles, without which, our noble calling must prove a failure. As you have given evidence of your devotion to *medical ethics*, let me entreat you to continue thus to labor with the assurance that your labour will not be in vain. It is true that, in medicine, as politics, we have among us men who are pregnant with *tricks* and *rings*, and are ever ready to bring these to bear, for the purpose of making them subserve their debasing purposes. The time has come when such men must be brought out before the light of truth, and learn that they cannot be sustained so long as they are influenced by such motives. We want to see our associations and medical organizations based upon the principles of truth, and their membership composed alone of such men in the Profession, who love the truth and hold it as paramount to every other consideration."

We thank our friend for his words of cheer and encouragement. We have, in the past, endeavord in the conduct of our JOURNAL, to make it useful to the profession; 1st., by condensation of matter, and 2d, by advocating the loftiest principles of the Profession. We are not aware that any one has taken issue with us upon the second point,—at least not openly; for to do so, would be to condemn those principles which all desire to have appear they endorse. The trouble with those who violate ethics is, that they do so covertly, avowing all the time their admiration for them. But by their fruits they shall be known. Our Journal, while being devoted to science will not overlook other interests of the Profession. Hence it will discuss principles, and will attempt to show the Profession, by holding up the law (as a mirror), what it takes to constitute a medical man, a first-class gentlemen and physician; for, whenever a man is found who wilfully violates and ignores medical law, the Profession has "the measuring-stick" by which, at once, he may fix beyond doubt, the status of such an individual, despite his vociferous assertions of truth and honor, and the advocacy of him by over-partial friends. No man can successfully cover up the misdeeds of his friend. The attempt to do so will fix indelibly upon him the stain of moral cowardice and unreliability; for he, cowardly, quails before the faults of his friends rather than make the effort to correct them, and becomes as guilty, morally, when he, by his advocacy of him, endorses them. Not only does he display moral cowardice and unrelibility, but, by such acts, he makes the law, so far as he can, valueless. We have such men every where; in Church, State, Politics, Medicine; foul, cancerous growths, contaminating society and bringing shame and reproach (and oftentimes destruction) upon every organization to which they cling. Society can never be pure until the law, human and Divine, shall be obeyed. When it condemns, man to cover his own folly or to cloak that of his friend, cannot stay the moral power of its sentence. Like the silly King, he may sit enthroned in his folly vainly bidding the sea to roll away at his command. To make a pure society the law must be pure; and to make a pure character—one which shall command the confidence and respect of all—the law must clothe such an one in its garments of purity; and when so clothed that man is invulnerable against the shafts of his enemies.

We are pleased that our friend—with many others who have written us—so heartily endorses our plan in the conduct of our Journal; especially the objects we have in view by associating with us in the management of the Companion, the gentlemen who are now its associate Editors. That these gentlemen will give tone and character to the Journal and be the means of furnishing the profession of the South a first-class Journal we ardently wish, and confidently expect. We trust, therefore, that every professional man of this and adjoining States will come up to our support,—fortified as we are by the very best medical intellects of our section—and make the Companion the journal of the South.

